# Match and training injury incidence in rugby league: A systematic review, pooled analysis, and update on published studies

**DOI:** 10.1016/j.smhs.2022.03.002

**Published:** 2022-03-27

**Authors:** Doug A. King, Trevor N. Clark, Patria A. Hume, Karen Hind

**Affiliations:** a Sports Performance Research Institute New Zealand (SPRINZ), Faculty of Health and Environmental Science, Auckland University of Technology, Auckland, New Zealand; b Traumatic Brain Injury Network (TBIN), Auckland University of Technology, Auckland, New Zealand; c School of Science and Technology, University of New England, Armidale, NSW, Australia; d Faculty of Sport, Event Management, Tourism and Hospitality, International College of Management Sydney, Manly, NSW, Australia; e Wolfson Research Institute for Health and Wellbeing, Department of Sport and Exercise Sciences, Durham University, Durham, United Kingdom

**Keywords:** Rugby league, Injury epidemiology, Match, Training, Pooled analysis, Concussion

## Abstract

In studies reporting rugby league injuries, match injuries varied depending upon participation level. To review and update pooled data estimates for rugby league injury epidemiology and add information for participation levels in match and training environments. A systematic review and pooled analysis for published studies reporting rugby league match and training injuries. Searches were performed in the PubMed, CINHAL, ScienceDirect, Scopus, SPORTDiscus, SpringerLink, and Wiley Online databases. Studies were considered if they reported on rugby league match or training injuries between Jan 1990 to June 2021. Two authors (DK, TC) extracted the study characteristics, numerical data and assessed the article quality, by adhering to the protocol for systematic review of observational studies (MOOSE) and the STrengthening and Reporting of OBservational studies in Epidemiology (STROBE**)** statement. The 46 studies included a combined exposure of 419,037 h and 18,783 injuries incorporating 158,003 match-hr and 15,706 match injuries (99.4 [95%*CI*: 97.9–101.0] per 1000 match-hr) and 264,033 training-hr and 3077 training injuries (11.8 [95%*CI*: 11.4–12.2] per 1000 training-hr). Of included studies, 47.9% utilised a medical attention/treatment injury definition. There was a five-fold difference in injuries for the semi-professional participation level (431.6 per 1000 match-hr) compared with professional (*RR*: 4.92; *p* < 0.001) and elite (*RR*: 3.77; *p* < 0.001) participation levels. The hooker recorded the highest pooled injury incidence (93.1 per 1000 match-hr). Compared to the 2014 analysis there was a 10-fold increase for head-neck region (*RR*: 10.7; *p* < 0.001) injury incidence, and more injuries for the ball carrier (*RR*: 1.1; *p* = 0.008) and tackler (*RR*: 1.2; *p* = 0.001). There was a three-fold decrease in injury incidence in the first half (*RR*: 2.9; *p* < 0.001) and a two-fold decrease in the second half (*RR*: 2.3; *p* < 0.001) of matches. While rugby league match and training injury incidence had decreased since 2014, the increase in head injuries, and greater injury rate at the semi-professional level, mean further injury prevention interventions are needed.

## Highlights

• Based on 46 studies, updated estimates of injury incidence for rugby league-related activities ranged from 14.6 per 1000 match-hr (Junior) to 431.6 per 1000 match-hr (Semi-Professional) participation levels for match activities and 5.3 per 1000 training-hr (Professional) to 97.1 per 1000 training-hr (Elite) participation levels for training activities.

Compared with the pooled analysis in 2014, the current analysis showed:

• The hooker playing position continues to have the highest match injury risk (93.1 per 1000 match-hr).
• The lower limb (64.5 per 1000 match-hr) continues to be the body region with the highest injury risk.
• The quadriceps was the most reported injury site (28.5 per 1000 match-hr).
• The incidence of concussion in studies reporting on rugby league match injuries increased (7.7 vs. 11.6 per 1000 match-hr); and
• Most concussions took 28 days to recover, which conflicts with the Concussion in Sport Consensus Statement that reported 80%–90% of all concussions recover in seven to ten days.

## Abbreviations

*CI*: Confidence Interval
hr: hour
MOOSE: Meta-analysis Of Observational Studies in Epidemiology
mTBI: mild Traumatic Brain Injury
PRISMA: Preferred Reporting Items for Systematic Reviews and Meta-Analyses
PROSPERO: Prospective Register of Systematic Reviews
RR: Risk Ratio
SPSS: Statistical Package for Social Sciences
STROBE: STrengthening and Reporting of OBservational studies in Epidemiology

## Introduction

Rugby league is an international collision sport, with junior, amateur, semi-professional and professional levels of competition.^1^ It is a challenging contest comprising of intense bouts of activity such as tackling and sprinting, interspersed with short bouts of lower intensity activities such as jogging and walking.^2^ Individual players undergo an average of 29–55 physical collisions (tackles and ball-carries) per game.^3^ Rugby league has a high incidence of injury, especially when compared with other collision sports like rugby union,^4^ as a consequence of the physical collisions.^5^ As a result, there is a constant risk of injuries occurring^6^ and the injury incidence varies depending upon the participation level^5^ for both match (1^7^ to 825^5^^,^^8^ per 1000 match hr) and training (12.2^9^ to 105.8^10^ per 1000 training hr) injuries. A previously utilised strategy^11^ is to combine the information provided by epidemiological studies into a single estimate,^12^^,^^13^ termed a pooled analysis.^14^ To achieve this technique, it is recommended that all studies included in the analysis be on common grounds, have similar definitions, have a comparable population, and have adequate and specific exposure data.^15^ By pooling the data, the information provided can then be statistically re-analysed providing more precise injury data.^12^ Pooled analysis has been applied previously to rugby league injury epidemiological studies.^11^^,^^16^, ^17^, ^18^ This approach has been reported to produce an overall estimation of injuries recorded by incorporating data provided by the studies utilised.^19^ The limitations of a pooled analysis methodology have been described elsewhere,^12^^,^^20^ and they include differences in: study design (observation vs. self-reported injury);^21^^,^^22^ injury type, site and severity definitions; data collection methods and times; data recording medium and the maintenance of the data medium; identification of data utilised. Despite these limitations, the strength of a pooled analysis is that it provides more accurate estimates of injury rates than the individual studies that provided the data.^11^ Therefore, it can be utilised to compare against other pooled studies to obtain a combined estimator of the quantitative effect of the relative risk of injuries in rugby league match and training activities.^12^^,^^23^

The purpose of this study was to review and update the pooled data estimates on rugby league injury epidemiology and to provide additional information for semi-professional, amateur, and junior participation levels in both match and training environments. Specifically, this included estimates of injury incidence, injury severity, site and type, and the comparison of injury rates for player positions and for the different rugby league participation levels.

## Methods

The methodology utilised in this pooled analysis was similar to previous pooled analysis studies reporting rugby league injuries^11^^,^^17^^,^^18^ and followed steps described by Friedenreich^23^ and Blettner et al.^24^ An additional advantage to utilising a pooled analysis approach is that the same statistical model can be utilised with data from methodologically diverse studies.^25^ The review was submitted with the International Prospective Register of Systematic Reviews (PROSPERO) on 3^rd^ July 2021 (CRD42021265640). Guidelines for reporting systematic reviews (PRISMA: Preferred Reporting Items for Systematic Reviews and Meta-Analyses^26^) and observational studies (MOOSE: Meta-analysis Of Observational Studies in Epidemiology^27^) were followed. The PRISMA and MOOSE guidelines contain checklists that were utilised for conducting and reviewing the included studies.

**Fig. 1:**
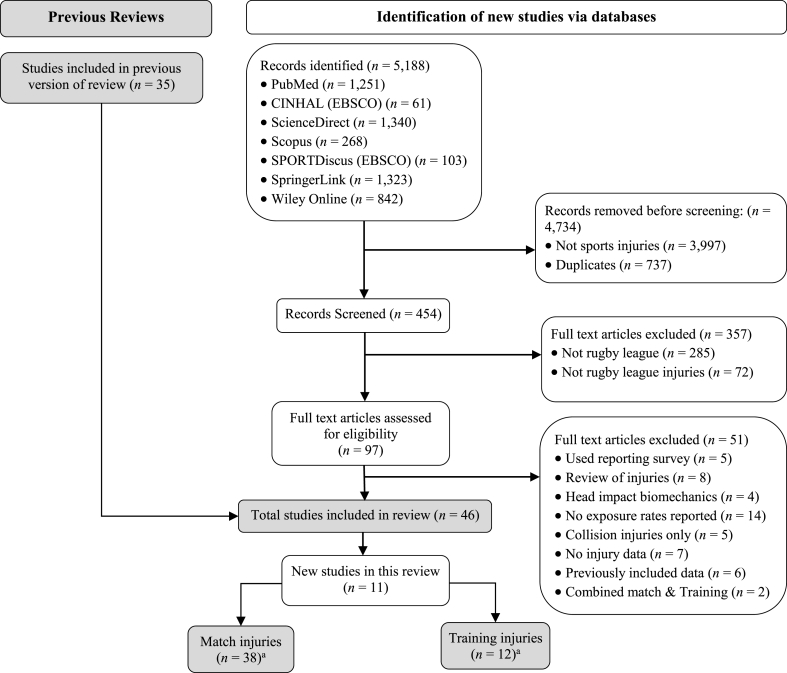
PRISMA 2020^26^ flow diagram for the identification, screening, eligibility, and inclusion for the literature reporting on rugby league match and training injuries.

Articles were identified from an initial search of the online databases from January 1990 to June 2021 (see Fig. 1). The search was undertaken with the key search terms of ‘Rugby League’, ‘rugby’, ‘league’ AND (Match OR Training) AND (injury OR injur∗ OR athletic injuries OR athlet∗ injur∗) AND (incidence OR epidemiology OR epidemiol∗ OR "injury incidence" OR injury rate). The reference lists of those articles retrieved for inclusion in this review were also hand-searched to identify any other relevant articles. Key articles were retrieved via online databases and through hand-searching reference lists and these were also used for further searches using the Web of Science Cited Reference function. During the second stage of the literature search, the titles and abstracts of articles were reviewed to assess eligibility for inclusion in this review.

To establish some control over the heterogeneity of the studies,^27^ the following inclusion criteria were established. Published studies that reported the incidence of injury in rugby league match and training activities were collated and included in the pooled analysis if they met the following criteria: (a) Available in English; (b) Reported the match or training time exposure enabling calculation of rugby league player time injury rates; and (c) Reported injuries as a result of match or training injuries. Studies were excluded from this review if it was identified that the publication: (a) Was unavailable in English; (b) Did not provide match or training exposure enabling calculation of player time rates; (c) Did not report on injuries that occurred because of match or training activities; (d) Combined male and female sex, match or did not differentiate between match and training exposure and; (e) Was a case study; (f) Utilised previously published data on concussions; (g) Was a meta-analysis or systematic review of rugby league injuries; and (h) Conference abstracts or Theses.

All references were downloaded into a dedicated EndNote library (Endnote, X9.3.3; Clarivate Analytics, Philadelphia, PA, United States of America). The library was reviewed, and duplicate records were identified and removed. All publications identified were initially screened by publication title and abstract to identify eligibility. The full-text versions of the remaining articles were then retrieved and evaluated against the inclusion criteria. Those studies meeting the inclusion criteria were included in the review. The references of all relevant articles were searched for further articles. In cases of discrepancies of eligibility, another author assessed the publication to screen for eligibility.

Two authors (DK, TC) extracted the study characteristics, numerical data and assessed the quality, by adhering to the protocol for systematic review of observational studies (MOOSE).^27^ This approach enabled a more precise estimate of the effects of influential factors and considered confounding factors (i.e., participation level and age) and the heterogeneity of the studies.^25^

All included studies were independently assessed by two authors (DK, TC) reporting on the article quality utilizing the STrengthening and Reporting of OBservational studies in Epidemiology (STROBE) statement.^28^ The statement provides a 22-item checklist guidance on the reporting of observational studies in order to facilitate a critical appraisal of the study and for the interpretation of the results. Following the appraisal, the included studies were categorised as either poor, moderate, or good quality based on the percentage of fulfilled items on the STROBE checklist, with cut-off values of <50%, 50%–80%, and >80% respectively.^29^ Any disagreements in the scores were discussed between the authors to identify a mutual rating.

Those studies meeting the inclusion criteria underwent data extraction for information pertaining to participation level, and injury definition utilised, reported injuries and player position/group.

Not all studies included in the systematic review and pooled analysis utilised the standardized method for injury reporting (i.e., per 1000 match or training hr of exposure). As a result, calculations were required to convert some study data to the standardized method for injury reporting to enable a pooled analysis to be conducted. Authors were contacted for further information on some papers^30^, ^31^, ^32^ to enable these calculations to be conducted. If the authors did not respond,^30^, ^31^, ^32^ the data were excluded from the review and pooled analysis.

To enable meaningful comparisons, the sports injury definitions of the included studies were categorised into broad groups.^33^ These groups consisted of: (1) *Medical attention/treatment injury* (any injury that requires the assistance of sports medicine personnel with or without time loss from training or completion); (2) *Full-inclusive time-loss injury* (any injury that results in time lost from the competition and/or training); (3) *Semi-inclusive time-loss injury* (any injury that results in time lost from competition only); and (4) *All-inclusive injury* (an injury that requires the assistance of sports medicine personnel and/or that which results in time loss from the competition and/or training).

All studies were reviewed for any concussion/mild traumatic brain injury (mTBI) definition. Studies that only reported on concussions in rugby league were included in the review if they provided match or training exposures hours to enable a pooled analysis. Any definitions that were utilised were compared.

To enable meaningful comparisons, the sports injury definitions of the included studies were categorised into broad groups.^34^ These groups consisted of: (1) *Transient* (Injuries that result in no matches/training activities missed); (2) *Minor* (Injuries that result in one match/one week of training activities missed); (3) *Moderate* (Injuries that result in two to four matches/two to four weeks of training activities missed); and (4) *Major* (Injuries that result in five or more matches/five or more training activities missed).

Included studies were categorized based on the participation level^34^ as reported in the study. The categories included were: (1) *Junior* (up to age 12 yr played under a mini-modified or modified rules version of rugby league); (2) *Amateur* (played under rugby league international competition rules but not receiving any payment for participation); (3) *Semi-Professional* (from 16 yr old and playing under international competition rules and receiving moderate remuneration for participation as well as additional employment to generate income); (4) *Elite* (playing under international competition rules and receiving remuneration for participation and/or some additional employment); and (5) *Professional* (from 18 yr old and playing under international competition rules and receiving remuneration for participation as the primary source of income) levels of participation.

A combined estimate of injuries within a specific sport through pooled analysis^14^ provides more precise evidence and meaningful information about the sport, whilst controlling for between-study variation due to individual sub-cohort characteristics.^24^ A pooled analysis of rugby league studies that reported injuries was undertaken for studies where homogeneity occurred in terms of the injury definition utilised and the reporting of injury incidence was per 1000 match or training-hr. This strategy has been previously utilised in rugby-15s,^35^^,^^36^ rugby league^11^^,^^17^^,^^18^ and women's rugby union^37^ epidemiological studies to combine the information provided into a single estimate.^13^^,^^24^

The pooled calculation of the incidence of concussion was undertaken to report the incidence per 1000 h and 95% confidence intervals (*CI*).^38^ To compare between injury rates, risk ratios (*RR*s) were used. To test for significant differences between studies and player positions, chi-squared (*χ*^*2*^) goodness-of-fit tests were utilised. All statistics were carried out using the SPSS (IBM SPSS Statistics for Windows, Version 22.0, Armonk, NY: IBM Corp) statistical software packages. Statistical significance was set as *p < *0.05*.*

## Results

Of the 46 studies included in this review (see Fig. 1) 38 studies reported injuries that occurred during match activities (4 junior,^7^^,^^39^, ^40^, ^41^ 13 amateur,^42^, ^43^, ^44^, ^45^, ^46^, ^47^, ^48^, ^49^, ^50^, ^51^, ^52^, ^53^, ^54^ 5 semi-professional,^2^^,^^8^^,^^10^^,^^55^^,^^56^ 3 elite,^4^^,^^57^^,^^58^ 13 professional^3^^,^^9^^,^^16^^,^^59^, ^60^, ^61^, ^62^, ^63^, ^64^, ^65^, ^66^, ^67^, ^68^) and 12 studies reported injuries that occurred during training activities (3 amateur,^50^^,^^69^^,^^70^ 3 semi-professional,^8^^,^^10^^,^^71^ 2 elite,^72^^,^^73^ 4 professional^16^^,^^74^, ^75^, ^76^). Four studies reported on both match and training injuries.^8^^,^^16^^,^^51^^,^^69^ When pooled, there was a combined exposure of 406,184 exposure hr and 17,455 injuries. The 157,291 match-hr and 15,815 reported match injuries resulted in an injury incidence of 89.2 (95%*CI*: 87.7–90.7) per 1000 match-hr. The 264,033 training-hr and 3077 reported training injuries corresponded to an injury incidence of 11.8 (95%*CI*: 11.4–12.2) per 1000 training-hr. All the studies included in the pooled analysis were observational in design.

Studies that were reviewed but not included in this analysis only reported on collision injuries,^77^, ^78^, ^79^, ^80^, ^81^ did not report match exposure hr,^7^^,^^30^, ^31^, ^32^^,^^82^, ^83^, ^84^, ^85^, ^86^, ^87^, ^88^, ^89^, ^90^ did not report injury data,^57^^,^^77^^,^^91^, ^92^, ^93^ reported head impact biomechanics,^94^, ^95^, ^96^, ^97^ reported data already included in this review,^98^, ^99^, ^100^ utilised a survey questionnaire,^101^, ^102^, ^103^ were reviews,^1^^,^^6^^,^^11^^,^^17^^,^^18^^,^^104^, ^105^, ^106^ or reported on self-reported injuries.^107^^,^^108^

Nearly half (45.7%) of included studies^2^^,^^8^, ^9^, ^10^^,^^16^^,^^39^^,^^40^^,^^43^, ^44^, ^45^, ^46^, ^47^^,^^56^^,^^63^, ^64^, ^65^^,^^68^, ^69^, ^70^, ^71^, ^72^ utilised a medical attention/treatment injury definition. Nearly a quarter (23.9%) of studies^4^^,^^48^^,^^49^^,^^53^^,^^55^^,^^57^^,^^58^^,^^66^^,^^74^, ^75^, ^76^ utilised a full-inclusive time-loss injury definition, and 4.3% of studies^54^^,^^59^ utilised an all-inclusive injury definition. A quarter (26.1%) of included studies utilised a semi-inclusive injury definition,^3^^,^^7^^,^^60^, ^61^, ^62^^,^^73^ reported no injury definition^41^^,^^42^^,^^50^, ^51^, ^52^ or reported a medical attention/treatment injury definition^67^ and a modified concussion definition. Four included studies^41^^,^^50^, ^51^, ^52^ utilised the concussion in sports consensus statement definition.

The pooled injury incidence of 89.2 (95%*CI*: 87.7–90.7) per 1000 match-hr varied by participation level (see Table 1). There was a five-fold difference in injuries recorded at the semi-professional participation level (431.6 [95%*CI*: 417.2–446.5] per 1000 match-hr) when compared with professional (*RR*: 4.92 [95%*CI*: 4.77–5.08]; *p* < 0.001) and elite (*RR*: 3.77 [95%*CI*: 3.47–3.09]; *p* < 0.001) participation levels.

**Table 1:** Gender and participation level for match and training injuries by number of studies, number of participants, injuries recorded, exposure hours and injury incidence with 95% confidence interval for included published rugby league studies.

Activity	Gender/Participation level	Studies	Participants	Injuries	Match exposure	Injury Incidence
		*n*	*n*	*n*	hr	Rate (95%*CI*)
Match Injuries^a^						
Gender						
	Male^2^, ^3^, ^4^^,^^7^, ^8^, ^9^, ^10^^,^^16^^,^^39^, ^40^, ^41^, ^42^, ^43^, ^44^, ^45^^,^^47^, ^48^, ^49^, ^50^, ^51^, ^52^, ^53^, ^54^, ^55^, ^56^, ^57^, ^58^, ^59^, ^60^, ^61^, ^62^, ^63^, ^64^, ^65^, ^66^, ^67^, ^68^	37	4349	15,605	157,673	99.0 (97.4–100.5)
	Female^46^	1	100	101	329	306.8 (252.4–372.9)
Participation level						
	Professional^3^^,^^4^^,^^9^^,^^16^^,^^59^, ^60^, ^61^, ^62^, ^63^, ^64^, ^65^, ^66^, ^67^, ^68^	14	1209	9,044^bcde^	103,144	87.7 (85.9–89.5)
	Elite^57^^,^^58^	2	266	559^acde^	4881	114.5 (105.4–124.4)
	Semi-Professional^2^^,^^8^^,^^10^^,^^55^^,^^56^	5	663	3,346^abde^	7752	431.6 (417.2–446.5)
	Amateur^42^, ^43^, ^44^, ^45^, ^46^, ^47^, ^48^, ^49^, ^50^, ^51^, ^52^, ^53^, ^54^	13	2144	2,362^abce^	15,918	155.4 (149.3–161.8)
	Junior^7^^,^^39^, ^40^, ^41^	4	167	395^abcd^	27,028	14.6 (13.2–16.1)
	**Total**	**38**	**4449**	**15,706**	**158,003**	**99.4 (97.9–101.0)**
Training Injuries^b^						
Gender						
	Male^8^^,^^10^^,^^16^^,^^50^^,^^68^, ^69^, ^70^, ^71^, ^72^, ^73^, ^74^, ^75^, ^76^	12	1429	3077	264,033	11.7 (11.2–12.1)
Participation Level						
	Professional^16^^,^^68^^,^^74^, ^75^, ^76^	4	162	755^bcd^	199,877	3.8 (3.6–6.1)
	Elite^72^^,^^73^	2	403	1,022^acd^	10,528	97.1 (91.3–103.2)
	Semi-Professional^8^^,^^10^^,^^71^	3	295	1,116^abd^	22,268	50.1 (47.3–53.1)
	Amateur^50^^,^^69^^,^^70^	3	569	184^abc^	31,360	5.9 (5.1–6.8)
	**Total**	**12**	**1429**	**3077**	**264,033**	**11.7 (11.2–12.1)**

*CI*: Confidence Interval; (a) = per 1000 match-hr; (b) = per 1000 training-hr; Significant difference (*p* < 0.05) than (a) = Professional; (b) = Elite; (c) = Semi-Professional; (d) = Amateur; (e) = Junior.

Forwards (172.2 [95%*CI*: 165.9–178.8] per 1000 match-hr) recorded a higher pooled injury incidence than backs (*RR* = 1.36 [95%*CI*: 1.29–1.43], *p* < 0.001) (see Table 2). Semi-professional backs recorded more injuries (276.9 [95%*CI*: 259.9–294.9] per 1000 match-hr) than professional (RR: 5.22 [95%*CI*: 4.72–5.77]; *p* < 0.001) backs. The hooker recorded the highest pooled injury incidence (93.1 [95%*CI*: 76.8–113.0] per 1000 match-hr) (see Supplementary. Table 1) but this varied by participation level. The prop forward recorded the highest injury incidence (159.8 [95%*CI*: 126.7–201.7] per 1000 match-hr) at the amateur level. The fullback at the amateur level (157.6 [95%*CI*: 113.1–219.5] per 1000 match-hr) recorded more injuries when compared with the semi-professional (RR: 1.67 [95%*CI*: 1.07–2.61]; *p* = 0.034) and professional (*RR*: 3.55 [95%*CI*: 2.15–5.87]; *p* < 0.001) participation levels. Hit-Up Forwards recorded the highest injury incidence at the Junior level (286.3 [95%*CI*: 200.2–409.5] per 1000 match-hr) compared with the semi-professional (*RR*: 5.62 [95%*CI*: 3.85–8.22]; *p* < 0.001) and professional (*RR*: 8.61[95%*CI*: 5.88–12.60]; *p* < 0.001) participation levels.

**Table 2:** Summary of pooled analysis of match injuries by total and participation level per 1000 match-hr with 95% confidence intervals and number of studies by player role, injury region, top four reported injury type and injury cause, injury severity and injury period for included published rugby league studies.

	Total	Professional	Elite	Semi-Professional	Amateur	Junior
	Rate (95%*CI*) *n*	Rate (95%*CI*) *n*	Rate (95%*CI*) *n*	Rate (95%*CI*) *n*	Rate (95%*CI*) *n*	Rate (95%*CI*) *n*
**Player Role**						
Forwards	172.2^g^ (165.9–178.8) 19	77.4^bcdeg^ (71.6–83.8) 5	91.9^acdeg^ (72.8–115.9) 1	382.7^abdeg^ (361.1–405.6) 3	198.2^abcg^ (186.1–211.2) 8	235.4^abc^ (170.6–324.9) 1
Backs	127.8^f^ (122.7–133.0) 19	53.1^cdef^ (48.6–57.9) 5	42.1^cdef^ (30.7–57.9) 1	276.9^abdef^ (259.9–294.9) 3	158.3^abcf^ (148.3–169.1) 8	185.4^abc^ (132.5–259.5) 1
**Injury region**						
Head-Neck	37.8 (36.4–39.3) 32	29.1^bcde^ (27.4–30.8) 9	7.4^acde^ (5.3–10.2) 2	68.6^abde^ (63.0–74.7) 5	53.3^abce^ (49.8–57.1) 13	15.1^abcd^ (10.1–22.8) 3
Upper Limb	32.8 (31.3–34.3) 25	25.6^bcd^ (23.9–30.8) 7	12.8^acde^ (10.0–16.3) 2	72.8^abde^ (67.0–79.0) 5	31.4^abc^ (28.5–34.7) 9	23.7^bc^ (17.0–33.2) 2
Lower Limb	65.6 (63.5–67.8) 25	54.3^bcde^ (51.7–56.9) 7	27.3^acde^ (23.1–32.3) 2	157.2^abde^ (148.7–166.3) 5	50.0^abc^ (46.2–54.1) 9	38.4^abc^ (29.5–50.0) 2
Chest-Back-Other	16.3 (15.3–17.4) 25	15.4^bde^ (26.5–30.5) 8	5.2^ace^ (3.5–7.6) 2	17.8^be^ (15.1–21.0) 5	20.8^ae^ (18.4–23.5) 9	11.2^abcd^ (6.8–18.2) 2
**Injury type**						
Haematomas	44.1 (41.6–46.7) 13	9.8^bcde^ (8.0–12.1) 2	12.2^acd^ (9.5–15.6) 2	137.5^abde^ (127.7–148.0) 3	44.7^abce^ (11.5–26.5) 3	14.0^acd^ (9.0–21.6) 2
Contusion	38.3 (36.5–40.1) 20	21.5^bcde^ (19.9–23.3) 7	8.4^acd^ (5.8–12.1) 1	80.5^abde^ (74.3–87.2) 3	100.0^abce^ (91.9–108.9) 7	5.6^acd^ (2.8–11.2) 2
Strain	39.2 (37.6–40.9) 26	20.5^bcde^ (19.3–21.7) 9	9.2^acd^ (6.9–12.2) 2	110.0^abde^ (102.7–117.7) 4	44.3^abce^ (40.7–48.1) 9	11.2^acd^ (6.8–18.2) 2
Sprain	37.2 (35.7–38.8) 27	22.2^bcde^ (21.0–23.5) 9	18.5^acde^ (15.1–22.7) 2	69.7^abde^ (63.9–75.9) 4	33.5^abc^ (30.4–36.8) 10	32.1^abc^ (24.1–42.9) 2
**Injury Cause**						
Ball Carrier	58.2 (55.8–60.7) 25	24.6^cd^ (22.6–26.9) 7	16.5^cd^ (12.6–21.4) 1	117.4^abde^ (110.0–125.3) 5	140.3^abce^ (130.3–151.1) 10	19.5^cd^ (13.0–29.3) 2
Tackler	36.3 (34.5–38.3) 25	14.2^cd^ (12.6–15.9) 7	13.2^cd^ (9.8–17.7) 1	78.2^abe^ (72.2–84.6) 5	82.7^abe^ (75.1–91.0) 10	13.6^cd^ (8.3–22.1) 2
Collision Player	35.8 (32.7–39.2) 10	N/R	3.6^cde^ (2.0–3.3) 1	55.1^bde^ (50.1–60.6) 5	16.5^bc^ (9.4–29.0) 3	10.1^bc^ (5.6–1.8) 1
Collision Other	25.8 (23.1–28.7) 8	N/R	3.3^c^ (1.8–5.9) 1	41.5^bde^ (37.2–46.3) 5	2.2^c^ (0.6–8.9) 1	1.8^c^ (0.5–7.3) 1
**Injury Severity**						
Transient	114.7 (112.2–117.2) 21	70.3^bcd^ (68.2–72.5) 9	32.5^acde^ (24.6–42.8) 1	485.1^abde^ (467.3–503.5) 4	382.5^abce^ (358.2–408.5) 6	88.1^bcd^ (61.6–126.0) 1
Missed-Match	36.1 (34.7–37.5) 25	22.6^bcde^ (21.4–23.8) 9	210.4^acde^ (188.7–234.6) 1	78.4^adeb^ (71.5–86.1) 4	86.1^abc^ (78.3–94.7) 9	119.0^abc^ (90.5–156.6) 2
**Injury period**						
1st half	161.7^i^ (152.4–171.5) 11	N/R	N/R	90.2^dei^ (74.5–109.1) 1	175.3^ci^ (164.6–186.7) 9	211.5^c^ (152.5–293.1) 1
2nd half	183.3^h^ (173.5–193.7) 11	N/R	N/R	139.5^deh^ (119.7–162.6) 1	191.4^ch^ (180.2–203.3) 9	223.2^c^ (162.4–306.7) 1

*CI*: Confidence Interval; *n* = number of studies; N/R = not reported; Significant difference (*p* < 0.05) than (a) = Professional; (b) = Elite; (c) = Semi-Professional; (d) = Amateur; (e) = Junior; (f) = forwards; (g) = backs; (h) = 1st half; (i) = 2nd half.

The lower limb (65.6 [95%*CI*: 63.5–67.8] per 1000 match-hr) was the most reported pooled injury site with the quadriceps (28.5 [95%*CI*: 26.4–30.8] per 1000 match-hr) reported as the most injured injury location (see Table 2 and Supplementary Table 2). Semi-professional players recorded more injuries to the knee (54.7 [95%*CI*: 49.7–60.2] per 1000 match-hr) than players at the junior (*RR*: 2.80 [95%*CI*: 1.92–4.08]; *p* < 0.001), amateur (*RR*: 3.02 [95%*CI*: 2.56–3.57]; *p* < 0.001), professional (*RR*: 3.26 [95%*CI*: 2.85–3.73]; *p* < 0.001), and elite (*RR*: 7.22 [95%*CI*: 5.19–10.04]; *p* < 0.001) participation levels. Amateur players recorded a higher incidence of injury to the head (49.1 [95%*CI* 45.0–53.5] per 1000 match-hr) than players at the professional (*RR*: 1.73 [95%*CI*: 1.55–1.93]; *p* < 0.001), semi-professional (*RR*: 2.00 [95%*CI*: 1.66–2.40]; *p* < 0.001), junior (*RR*: 3.73 [95%CI: 2.39–5.81]; *p* < 0.001), and elite (*RR*: 7.46 [95%*CI*: 5.25–10.59]; *p* < 0.001) participation levels.

Hematomas (44.1 [95%*CI*: 41.6–46.7] per 1000 match-hr) were the most reported pooled injury type, but this varied by participation level (see Table 2 and Supplementary Table 3). Amateur players recorded more fracture-dislocations (18.5 [95%*CI*: 15.3–22.3] per 1000 match-hr) than semi-professional, (*RR*: 1.38 [95%*CI*: 1.03–1.85]; *p* = 0.034), elite (*RR*: 2.99 [95%*CI*: 2.01–4.45]; *p* < 0.001) and professional (*RR*: 1.59 [95%*CI*: 1.30–1.95]; *p* < 0.001) participation levels. There were fewer concussions reported at the elite (3.6 [95%*CI*: 2.3–5.7] per 1000 match-hr) than professional (*RR*: 2.47 [95%*CI*: 1.55–3.95]; *p* < 0.001), semi-professional (*RR*: 2.97 [95%*CI*: 1.75–5.04]; *p* < 0.001), amateur (*RR*: 3.08 [95%*CI*: 1.85–5.12]; *p* < 0.001), and junior (*RR*: 3.12 [95%*CI*: 1.61–6.03]; *p* < 0.001) participation levels.

The tackle was the most common injury cause with the ball carrier (58.2 [95%*CI*: 55.8–60.7] per 1000 match-hr) having the highest injury rate (see Table 2 and Supplementary Table 3). Amateur players recorded more injuries as the ball carrier (140.3 [95%*C*I: 130.3–151.1] per 1000 match-hr) than players at the semi-professional (*RR*: 1.20 [95%*CI*: 1.09–1.31]; *p* < 0.001), professional (*RR*: 6.94 [95%*CI*: 6.14–7.85]; *p < *0.001), junior (*RR*: 7.20 [95%*CI*: 4.78–10.85]; *p < *0.001) and elite (*RR*: 8.52 [95%*CI*: 6.50–11.18]; *p < *0.001) participation levels.

Players at the elite level recorded more missed match injuries (210.4 [95%*CI*: 188.7–234.6] per 1000 match-hr) than players at the junior (*RR*: 1.77[95%*CI*: 1.34–2.33]; *p* < 0.001), amateur (*RR*: 2.44 [95%*CI*: 2.14–2.79]; *p* < 0.001), semi-professional (*RR*: 2.68 [95%*CI*: 2.35–3.06]; *p* < 0.001) and professional (*RR*: 9.32 [95%*CI*: 8.35–10.41]; *p* < 0.001) participation levels (see Table 2 and Supplementary Table 3). Players at the semi-professional level (485.1 [95%*CI*: 467.3–503.5] per 1000 match-hr) recorded more transient injuries than players at the amateur (*RR*: 1.27 [95%*CI*: 1.20–1.34]; *p* < 0.001), junior (*RR*: 6.36 [95%*CI*: 5.93–6.83]; *p* < 0.001), professional (*RR*: 8.93 [95%*CI*: 8.56–9.32]; *p* < 0.001) and elite (*RR*: 34.81 [95%*CI*: 26.83–45.93]; *p* < 0.001) participation levels.

Not all participation levels reported injury match period (see Table 2 and Supplementary Table 3). There were more injuries reported in second (183.3 [95%*CI*: 173.5–193.7] vs. 161.7 [95%*CI*: 152.4–171.5] per 1000 match-hr) than the first half of matches (*RR*: 1.13 [95%*CI*: 1.05–1.22]; *p* = 0.002).

Training injuries varied by participation level (see Table 3). Players at the elite level (97.1 [95%*CI*: 91.3–103.2] per 1000 training-hr) recorded significantly more training injuries than players at the professional (*RR*: 22.18 [95%*CI*: 20.32–24.20]; *p* < 0.001), semi-professional (*RR*: 1.94 [95%*CI*: 1.79–2.10]; *p* < 0.001) and amateur (*RR*: 16.54 [95%*CI*: 14.16–19.33]; *p* < 0.001) participation levels.

**Table 3:** Summary of pooled analysis of training injuries by total and participation level per 1000 training-hr with 95% confidence intervals and number of studies by player role, injury region, top four reported injury type and injury cause, injury severity and injury period for included published rugby league studies.

	Total	Professional	Elite	Semi-Professional	Amateur
	Rate (95%*CI*) *n*	Rate (95%*CI*) *n*	Rate (95%*CI*) *n*	Rate (95%*CI*) *n*	Rate (95%*CI*) *n*
**Player Role**					
Forwards	9.9^f^ (9.0–10.9) 4	N/R	N/R	25.9^df^ (23.2–28.9) 1	3.2^c^ (2.6–3.9) 3
Backs	7.6^e^ (6.8–8.5) 4	N/R	N/R	19.3^de^ (17.0–22.0) 1	2.6^c^ (2.1–3.2) 3
**Injury region**					
Head-Neck	0.8 (0.7–0.9) 10	0.3^bcd^ (0.2–0.4) 2	8.8^acd^ (7.2–10.8) 2	1.8^abd^ (1.3–2.4) 3	0.7^abc^ (0.5–1.1) 3
Upper Limb	1.0 (0.9–1.2) 9	0.2^bcd^ (0.2–0.3) 2	9.6^acd^ (7.9–11.7) 2	2.4^ab^ (1.9–3.2) 3	2.8^ab^ (2.0–3.9) 2
Lower Limb	8.1 (7.7–8.5) 9	10.6^bc^ (9.4–11.9) 2	65.8^acd^ (61.1–70.9) 2	36.2^abd^ (33.7–38.7) 3	8.6^bc^ (7.2–10.4) 2
Chest-Back-Other	1.7 (1.6–1.9) 9	0.7^bc^ (0.6–0.8) 2	12.8^acd^ (10.8–15.2) 2	6.0^abd^ (5.0–7.1) 3	0.8^bc^ (0.4–1.4) 2
**Injury type**					
Abrasion	8.0 (6.9–9.3) 4	N/R	11.5^c^ (9.6–13.7) 2	4.3^b^ (3.2–5.8) 2	N/R
Blister	6.6 (5.3–8.4) 2	N/R	6.6 (5.3–8.4) 2	N/R	N/R
Strain	4.2 (4.0–4.5) 10	0.8^bcd^ (0.7–0.9) 3	25.9^ab^ (23.0–29.2) 2	23.2^ad^ (21.3–25.3) 3	5.0^abc^ (3.9–6.3) 2
Haematomas	2.7 (2.1–3.5) 5	0.3^bc^ (0.0–2.5) 1	3.4^a^ (2.5–4.7) 2	2.6^a^ (1.8–3.9) 2	N/R
**Injury Cause**					
Overexertion	17.9 (16.6–19.3) 5	N/R	33.4^cd^ (30.1–37.1) 2	20.5^bd^ (18.4–22.8) 2	0.1^bc^ (0.0–0.6) 1
Contact	13.9 (10.5–18.3) 1	N/R	N/R	13.9 (10.5–18.3) 1	N/R
Collision Other	8.6 (7.2–10.3) 3	N/R	8.7 (7.1–10.7) 2	8.2 (5.7–11.7) 1	N/R
Fall	8.3 (7.3–9.5) 4	N/R	10.6^c^ (8.8–12.8) 2	6.8^d^ (5.6–8.2) 2	N/R
**Injury Severity**					
Transient	8.2 (7.8–8.5) 9	2.4^bcd^ (2.2–2.6) 3	83.4^acd^ (75.0–92.8) 1	46.7^abd^ (43.9–49.6) 3	4.6^abc^ (3.6–5.9) 2
Missed Match	2.0 (7.3–9.5) 10	1.0^bcd^ (0.9–1.2) 3	27.2^acd^ (22.5–32.7) 1	3.4^ab^ (2.7–4.3) 3	3.6^ab^ (3.0–4.3) 3
**Training Period**					
1st half	2.5^f^ (2.0–3.1) 3	N/R	N/R	N/R	2.5^f^ (2.0–3.1) 3
2nd half	3.3^e^ (2.8–4.1) 3	N/R	N/R	N/R	3.3^e^ (2.8–4.1) 3

*CI*: Confidence Interval; *n* = number of studies; N/R = not reported; Significant difference (*p* < 0.05) than (a) = Professional; (b) = Elite; (c) = Semi-Professional; (d) = Amateur; (e) = 1st half; (f) = 2nd half.

Forwards (9.9 [95%*CI*: 9.0–10.9] per 1000 training-hr) recorded more pooled injuries than backs (7.6 [95%*CI*: 6.8–8.5] per 1000 training-hr; *RR* = 1.31 [95%*CI*: 1.13–1.51], *p* < 0.001) (see Table 3). There were more injuries recorded by semi-professional forwards than amateur forwards (*RR*: 8.12 [95%*CI*: 6.50–10.16]; *p* < 0.001).

The most common pooled injury region was the lower limb (8.1 [95%*CI*: 7.7–8.5] per 1000 training-hr). Elite level participants (65.8 [95%*CI*: 61.1–70.9] per 1000 training-hr) recorded more lower limb training injuries when compared with those at the semi-professional (*RR* = 1.82 [95%*CI*: 1.65–2.01], *p* < 0.001), professional (*RR* = 6.20 [95%*CI*: 5.41–7.12], *p* < 0.001), and amateur (*RR* = 7.62 [95%*CI*: 6.26–9.28], *p* < 0.001) participation levels (Table 3). The quadriceps (10.9 [95%*CI*: 10.1–11.8] per 1000 training-hr) recorded the highest pooled injury site. There were more injuries recorded to the quadriceps at the elite (31.5 [95%CI: 28.3–35.1] per 1000 training-hr) than the semi-professional (*RR* = 2.02 [95%*CI*: 1.73–2.36], *p* < 0.001) and professional (*RR* = 20.37 [95%*CI*: 15.14–27.40], *p* < 0.001) participation levels (Supplementary Table 4). There were more injuries recorded to the head−neck body region at the elite (8.8 [95%*CI*: 7.2–10.8] per 1000 training-hr) than the amateur (*RR* = 4.62 [95%*CI*: 2.43–8.81], *p* < 0.001) and professional (*RR* = 9.81 [95%*CI*: 6.32–15.23], *p* < 0.001) participation levels (Supplementary Table 4).

Abrasions (8.0 [95%*CI*: 6.9–9.3] per 1000 training-hr) were the most common pooled injury type although this varied by participation level (see Table 3 and Supplementary Table 4). Amateur (5.0 [95%*CI*: 3.9–6.3] per 1000 training-hr) and semi-professional (23.2 [95%*CI*: 21.3–25.3] per 1000 training-hr) participants recorded strains as the most common injury type whereas the elite level recorded sprains (29.0 [95%*CI*: 25.9–32.4] per 1000 training-hr) and the professional level recorded other (3.6 [95%*CI*: 2.5–5.0] per 1000 training-hr) as the most common injury types. There were fewer concussions recorded at the professional level (0.01 [95%*CI*: 0.00–0.04] per 1000 training-hr) than at the amateur (*RR*: 37.09 [95%*CI*: 3.86–356.53]; *p* < 0.001), semi-professional (*RR*: 44.01 [95%*CI*: 2.75–703.50]; *p* < 0.001), and elite (*RR*: 76.79 [95%*CI*: 8.97–657.28]; *p* < 0.001) participation levels.

The most common pooled injury cause was overexertion (17.9 [95%*CI*: 16.6–19.3] per 1000 training-hr) (see Table 3). There were more overexertion injuries reported at elite (33.4 [95%*CI*: 30.1–37.1] per 1000 training-hr) than semi-professional (*RR*: 1.63 [95%*CI*: 1.41–1.90]; *p* < 0.001), and amateur (*RR*: 387.13 [95%*CI*: 54.39–3755.45]; *p* < 0.001) participation levels. More injuries were recorded as the tackler at the amateur (2.9 [95%*CI*: 2.1–4.0] per 1000 training-hr) than elite (RR: 1.91 [95%*CI*: 1.07–3.43]; *p* = 0.027), semi-professional (*RR*: 5.34 [95%*CI*: 1.29–22.11]; *p* = 0.01), and professional (*RR*: 134.22 [95%*CI*: 84.83–212.37]; *p* < 0.001) participation levels (see Supplementary Table 5).

There were more transient injuries at elite (46.7 [95%*CI*: 43.9–49.6] per 1000 training-hr) than semi-professional (*RR*: 1.79 [95%*CI*: 1.59–2.01]; *p* < 0.001), amateur (*RR*: 18.19 [95%*CI*: 13.85–23.88]; *p* < 0.001), and professional (*RR*: 34.66 [95%*CI*: 30.25–39.71]; *p* < 0.001) participation levels (see Table 3). The elite level recorded more moderate injuries (2.4 [95%*CI*: 1.3–4.5] per 1000 training-hr) than the amateur (*RR*: 2.46 [95%*CI*: 1.08–5.61]; *p* = 0.027), semi-professional (*RR*: 4.54 [95%*CI*: 1.96–10.50]; *p* = 0.001), and professional (*RR*: 11.10 [95%*CI*: 5.52–22.30]; *p* < 0.001) participation levels (see Supplementary Table 5).

Only the amateur level reported the training period when the injuries occurred (see Table 3). There were more injuries recorded in the second (3.3 [95%*CI*: 2.8–4.1] vs 2.5 [95%*CI*: 2.0–3.1] per 1000 training-hr) than the first half of training periods (*RR*: 1.36 [95%*CI*: 1.02–0.83]; *p* = 0.038).

Differences between the 2014 pooled analysis for rugby league^18^ and the current analysis are given in Table 4. There was a two-fold increase in the incidence of match injuries to forwards (*RR*: 2.3 [95%*CI*: 2.2–2.5]; *p* < 0.001) when compared to the previous analysis. There was a 10-fold increase in the incidence of injuries to the head-neck region (*RR*: 10.7 [95%*CI*: 10.1–11.3]; *p* < 0.001), more injuries were recorded by the ball carrier (*RR*: 1.1 [95%*CI*: 1.0–1.2]; *p* = 0.008) and the tackler (*RR*: 1.2 [95%*CI*: 1.1–1.4]; *p* < 0.001), and a three-fold decrease in the incidence of injuries in the first half (*RR*: 2.9 [95%*CI*: 2.7–3.1]; *p* < 0.001) and a two-fold decrease in the second half (*RR*: 2.3 [95%*CI*: 2.2–2.5]; *p* < 0.001) of matches when compared to the previous analysis.

**Table 4:** Summary of pooled analysis of current study with previous pooled analysis^18^ of rugby league match and training injuries by player role, injury region, injury type, injury cause, injury severity and injury period for injury incidence per 1000 h with 95% confidence interval for published rugby league studies.

	Match related injuries		Training related injuries	
	2021	2014^18^	2021	2014^18^
	Total	Total	Total	Total
	Rate (95%*CI*) *n*	Rate (95%*CI*) *n*	Rate (95%*CI*) *n*	Rate (95%*CI*) *n*
**Total IR**				
	89.2∗ (87.7–101.7) 38	147.6^#^ (145.1–150.2) 26	11.8∗ (11.4–12.2) 12	12.6^#^ (12.2–13.1) 11
**Player Role**				
Forwards	172.2∗ (165.9–178.8) 18	155.8^#^ (147.6–164.4) 10	9.9 (9.0–10.9) 4	8.5 (6.9–10.3) 2
Backs	127.8∗ (122.7–133.0) 18	112.4^#^ (106.0–119.2) 10	7.6 (6.8–8.5) 4	6.7 (5.3–8.3) 2
**Injury region**				
Head-Neck	37.8∗ (36.4–39.3) 32	34.4^#^ (32.8–36.1) 20	0.8 (0.7–0.9) 10	0.7 (0.6–0.9) 8
Upper Limb	32.8∗ (31.3–34.3) 25	34.6^#^ (32.8–36.5) 20	1.0 (0.9–1.2) 9	0.9 (0.8–1.0) 8
Lower Limb	65.9∗ (63.5–67.8) 25	69.1^#^ (66.6–71.7) 20	8.1∗ (7.7–8.5) 9	5.7^#^ (5.4–6.1) 8
Chest-Back-Other	16.3∗ (15.3–17.4) 25	18.3^#^ (17.0–19.7) 20	1.7∗ (1.6–1.9) 9	1.4^#^ (1.3–1.6) 8
**Injury type**				
Contusion	38.7∗ (37.0–40.6) 21	28.3^#^ (26.6–30.0) 17	1.3∗ (1.2–1.5) 9	2.5^#^ (2.3–2.7) 7
Strain	39.2∗ (37.6–40.9) 24	33.4^#^ (31.7–35.2) 17	4.2∗ (4.0–4.5) 10	3.6^#^ (3.4–3.9) 7
Sprain	37.2∗ (35.7–38.8) 25	27.5^#^ (25.9–29.2) 17	3.1∗ (2.9–3.4) 11	2.7^#^ (2.5–2.9) 6
Concussion	11.6∗ (10.8–12.5) 26	7.7^#^ (6.9–8.6) 18	0.1 (0.0–0.1) 6	0.1 (0.0–0.1) 6
**Injury Cause**				
Ball Carrier	58.2∗ (55.8–60.7) 25	49.0^#^ (45.6–52.5) 14	0.5∗ (0.4–0.6) 7	2.4^#^ (1.8–3.2) 4
Tackler	36.3∗ (34.5–38.3) 25	27.7^#^ (24.7–29.8) 14	0.5∗ (0.4–0.6) 6	2.0^#^ (1.4–2.7) 4
**Injury Severity**				
Transient	114.7∗ (112.2–117.2) 21	196.9^#^ (191.5–202.3) 14	8.2∗ (7.8–8.5) 9	7.4^#^ (7.0–7.8) 9
Missed-Match	36.1∗ (34.7–37.5) 25	70.2^#^ (67.5–72.9) 18	2.0∗ (1.8–2.1) 10	2.5^#^ (2.3–2.7) 9
**Injury period**				
1st half	161.7∗ (152.4–171.5) 11	461.3^#^ (438.0–485.7) 8	2.5∗ (2.0–3.1) 3	11.3^#^ (9.0–14.2) 2
2nd half	183.3∗ (173.5–193.7) 11	423.4^#^ (401.1–446.9) 8	3.3∗ (2.8–4.1) 3	15.4^#^ (12.7–18.8) 2

*CI*: Confidence Interval; IR = Injury Rate; *n* = number of studies; Significant difference (*p* ​< ​0.05) than (∗) 2014 pooled analysis; (^#^) = 2021 pooled analysis.

When reviewing training injuries (see Table 4) there were more training injuries recorded (*RR*: 1.5 [95%*CI*: 1.4–1.6]; *p* = 0.006) than the previous analysis. There were more injuries recorded to the lower limb body region (*RR*: 1.4 [95%*CI*: 1.3–1.5]; *p* < 0.001), more sprains (*RR*: 1.2 [95%*CI*: 1.2–1.3]; *p* = 0.006) there was a five-fold decrease (*RR*: 4.8 [95%*CI*: 3.4–6.8]; *p* < 0.001) for injuries to the ball carrier and a four-fold decrease (*RR*: 4.1 [95%*CI*: 2.8–6.0]; *p* < 0.001) for injuries to the tackler when compared with the previous analysis.

## Discussion

The aim of this review was to systematically update the incidence of injuries in rugby league as the previous study^18^ was published nearly 10 years ago. In doing so, we found that the incidence of rugby league injuries continues to vary by participation level from 14.6 per 1000 match-hr at the junior level to 431.6 per 1000 match-hr at the semi-professional level, with an overall pooled injury incidence of 99.4 per 1000 match-hr. This was similar to the previous pooled review^18^ with 14.4 per 1000 match-hr at the junior level of participation to 358.5 per 1000 match-hr at the semi-professional level. However, the overall injury incidence for that pooled analysis was notably higher at 147.6 per 1000 match-hr^18^ (see Table 4). This was similar for the training injuries with the recent study showing that professional level recording 3.8 per 1000 training-hr to 97.1 per 1000 training-hr at the elite level with an overall pooled injury incidence of 11.8 per 1000 training-hr. When compared with the previous study, there was a similar variation in the injury incidence with 3.8 per 1000 training-hr at the professional level to 65.2 per 1000 training-hr at the semi-professional level. The overall pooled injury incidence (11.8 per 1000 training-hr) was similar but there were meaningful differences observed.

There was a four-fold difference in the incidence of match injuries at the semi-professional compared with elite and professional participation levels. Pooled data analysis for matches indicated injuries occurred most to the hooker, from being tackled, and resulted in lower limb injury that was most commonly at the quadriceps, with haematomas and abrasions most common. There were more injuries in the 4th quarter of matches. For training injuries, there was a 22-fold increase in injury risk at the elite compared with the professional, and a 16-fold increase compared with the amateur participation levels. Overexertion (also termed cumulative trauma disorder)^109^ was the most common pooled injury cause, although there were significantly more (*p* < 0.001) reported at the elite level. There were more pooled transient than missed-match injuries reported at the elite level. Transient injuries accounted for 75.4% match and 79.4% training injuries.

Like previous studies^18^^,^^45^^,^^68^ transient injuries accounted for the majority of the match (75.4%) and training (79.4%) injuries. The inclusion of both missed-match and transient injuries in rugby league studies has been previously suggested^34^ but this also acts as a guide to the health care management and appointment of essential primary care personnel.^21^ Missed-match injuries still require assessment, management, and sometimes onward-referral to other health care service providers.^21^ This has resource implications on the healthcare practitioner when dealing with these injuries.^22^

The finding that the pooled concussion incidence was 11.6 per 1000 match-hr and 0.1 per 1000 training-hr was higher for the match (7.7 per 1000 match-hr) related injuries but lower (0.1 per 1000 training-hr) for training (0.3 per 1000 training-hr) related injuries than previous pooled analysis reviews.^17^^,^^18^ At the amateur level, the minimum stand-down period from a concussion was 21 days as stipulated by the national ruling body, and this was monitored in some studies using a saccadic reading application.^41^^,^^52^ It was reported that no player was able to return to training and/or match participation unless they had equalled or improved their reading time.^41^^,^^52^ As a result, some players took longer to recover from a concussion. This was similar in a recent study reporting on women's rugby union,^110^ where the mean missed-match duration for concussions was 28.9 ± 3.7 days and similar to a previous study^111^ where the majority of concussions took 28 days to recover. This finding is in conflict with the Concussion in Sport Consensus statement where it identified that 80%–90% of all concussions recover in seven to ten days.^112^^,^^113^ No study reported the concussion injury burden, yet this information may be useful in further studies to identify the burden of concussions on rugby league participants.

Although the incidence of concussion remained the same (0.1 per 1000 training-hr) in studies reporting on rugby league training, the incidence of concussion in studies reporting on rugby league match injuries increased between the two studies (7.7 vs. 11.6 per 1000 match-hr). This may be related to the increased awareness of this injury for all sporting codes.^114^ Despite attempts to standardise the definition of concussion through the Concussion in Sports Group, there have been several variations produced.^115^ Twenty-seven studies^4^^,^^10^^,^^16^^,^^17^^,^^30^^,^^39^, ^40^, ^41^^,^^43^^,^^46^, ^47^, ^48^, ^49^, ^50^, ^51^, ^52^, ^53^, ^54^, ^55^^,^^58^, ^59^, ^60^, ^61^, ^62^^,^^64^^,^^67^^,^^68^ reported concussions in match activities, and six studies^10^^,^^16^^,^^69^^,^^70^^,^^72^^,^^73^ reported concussions in training activities, only four of these studies^50^, ^51^, ^52^^,^^67^ provided a definition for concussion, and three of these^50^, ^51^, ^52^ utilised the Concussion in Sport Consensus definition. However, given that none of the other studies reported the concussion definition utilised, and there is no universal definition of concussion, the incidence of concussion may be higher than reported depending upon the methodological definitions utilised. Factors that need to be considered in regards to concussion reporting that may influence the results are the definition of a concussion (time loss vs. non-time loss), the knowledge of the people making the assessment, the availability of medical services to the team (as this will vary at the different participation levels) and the willingness of the player to report the signs of concussion.^115^

Three studies^30^, ^31^, ^32^ had identified limitations and unsuccessful attempts were undertaken to contact the authors for further clarification. Studies reported match and training injuries and calculated match player-hr but did not provide the exposure hr. By combining match and training data, there is a masking of differences between match and training injury exposure which renders inter-study comparisons meaningless.^33^ In addition, there was a notable concern with one study^31^ where the authors reported that they reclassified recorded concussions and descriptions of head injuries as suspected concussions. As a result, the data reported were not utilised in the pooled analysis for junior rugby league groups as there was no identification for match and training injuries and the number of reported concussions may have been different from what was reported by the team trainers. Future studies reporting on junior rugby league injuries need to consider separating match and training injuries and provide the reported injuries from the trainers involved.

The pooled analysis approach undertaken in this study produces an overall estimate of the injuries recorded by combining the data provided by the selected studies.^19^ As shown by this pooled analysis the incidence of injuries in rugby league is lower than the previous review and analysis of rugby league injuries (99.4 per 1000 match-hr) but like the previous study, the injury rate varied depending on the level of participation. The limitations with the use of a pooled analysis methodology have been previously described.^24^ Issues such as differences in study design (observation vs. self-reported injury);^116^ injury type, site and severity definitions; data collection methods and times; data recording medium and the maintenance of the data medium were considered and addressed through identification of the data utilised.^11^^,^^16^ An important issue in reporting rugby league injuries are the methodological approaches that each study utilised.^34^ Although there have been suggestions for the undertaking of epidemiological studies in rugby league, there remains a variation in the definitions utilised and this can limit interstudy comparisons.^34^ Epidemiological studies conducted at the semi-professional and professional levels of participation involve medical personnel such as medical doctors and physiotherapists while amateur and junior level participation studies typically do not have these personnel available.^17^ The variability of the medical providers available at the side-line may also influence the assessment of concussion as what may be a concussion to one person may not be to another.^117^ Despite these limitations, the strength of a pooled analysis is that it provides more accurate estimates of injury rates than the individual studies that provided the data.^11^ It can be utilised as comparisons against other pooled studies and to obtain a combined estimator of the quantitative effect of the relative risk of injuries in rugby league match and training activities.^23^^,^^24^

## Conclusions

The current pooled analysis examined a broad spectrum of published rugby league studies incorporating both match and training injuries at the professional, elite, semi-professional, amateur and junior levels of participation. The combined estimation of injuries within rugby league through a pooled analysis enables more precise evidence, and meaningful information about the injuries in match and training activities in rugby league. The finding that the incidence of rugby league injuries continued to vary by participation level with an overall pooled injury incidence of 99.4 per 1000 match-hr. This was similar for the training injuries with the recent study identifying an overall pooled injury incidence of 11.8 per 1000 training-hr. Pooled data analysis for matches indicated injuries occurred most to the hooker, from being tackled, and resulted in lower limb injury that was most commonly at the quadriceps, with haematomas and abrasions most common. There were more injuries reported in the 4th quarter of matches. The paucity of studies reporting on women's rugby league highlights an area of future research that is warranted. The inclusion of studies reporting specifically on concussions highlights the increasing awareness of this injury in sport and how the reporting can differ depending upon the injury definition and participation level. No study reported the concussion injury burden, yet this information may be useful in further studies to identify the burden of concussions on rugby league participants. Future studies reporting on rugby league injuries should consider incorporating a more detailed analysis of the activity, time, player position, and field position to assist with injury prevention programmes.

## Funding

No sources of funding were used to assist in the preparation of this article.

## Submission statement

As corresponding author, I hereby state that the manuscript has not been published previously, that it is not under consideration for publication elsewhere, that its publication is approved by all authors and tacitly or explicitly by the responsible authorities where the work was carried out, and that, if accepted, it will not be published elsewhere including electronically in the same form, in English or in any other language, without the written consent of the copyright-holder.

## Authors' contribution

Contributor statement: According to the definition given by the International Committee of Medical Journal Editors (ICMJE), the authors listed above qualify for authorship based on making one or more of the substantial contributions to the intellectual content of:

(i) Conception and design (DK, TC); and/or

(ii) Acquisition of data (DK); and/or

(iii) Analysis and interpretation of data (DK, TC, PH); and/or

(iv) Participated in drafting of the manuscript (DK, TC, PH, KH); and/or

(v) Critical revision of the manuscript for important intellectual content (DK, TC, PH, KH).

## Data availability statement

Data sharing is not applicable to this article as no datasets were generated or analysed during the current study.

## Conflict of interest

DK, TC, PH and KH declare that they have no conflicts of interest relevant to the content of this review.
