# Supplementary material for: Match and training injury incidence in rugby league: A systematic review, pooled analysis, and update on published studies
Source: Sports Med Health Sci. 2022 Mar 27;4(2):75–84. doi: 10.1016/j.smhs.2022.03.002 (PMC9219278; doi:10.1016/j.smhs.2022.03.002)
Supplement: Multimedia component 1 [file mmc1.docx]

**Supplementary Table 1:** Summary of pooled analysis of match injuries by total injuries recorded and participation level per 1,000 match-hrs. with 95% confidence intervals and number of studies by player position, player group and player role for included published rugby league studies.

|  |  | **Total** | **Professional** | **Elite** | **Semi-Professional** | **Amateur** | **Junior** |
| --- | --- | --- | --- | --- | --- | --- | --- |
|  |  | **Rate (95% CI)n** | **Rate (95% CI)n** | **Rate (95% CI)n** | **Rate (95% CI)n** | **Rate (95% CI)n** | **Rate (95% CI)n** |
| **Player Position** | |  |  |  |  |  |  |
|  | Prop | 85.9 (74.5-99.0)7 | 24.1^cde^ (16.3-35.7)1 | N/R | 118.0^a^ (94.8-147.0)2 | 159.8^a^ (126.7-201.7)3 | 267.3^a^ (158.3-451.3)1 |
|  | Hooker | 93.1 (76.8-113.0)8 | 48.2^cde^ (32.6-71.3)1 | N/R | 121.0^ae^ (89.1-164.3)2 | 144.1^a^ (101.9-203.7)4 | 190.9^ac^ (79.5-458.6)1 |
|  | Second Row Forward | 86.8 (75.4-100.0)8 | 42.4^cde^ (31.6-57.0)1 | N/R | 106.2^ae^ (84.3-133.8)2 | 135.1^ae^ (104.9-174.0)4 | 305.4^acd^ (187.1-498.6)1 |
|  | Loose Forward | 28.9 (22.6-37.0)7 | 5.8^cde^ (2.6-12.9)1 | N/R | 44.3^a^ (30.9-63.3)2 | 58.5^a^ (39.8-86.0)3 | 38.2^a^ (9.5-152.7)1 |
|  | Half-back | 49.7 (38.2-64.8)7 | 27.0^cde^ (16.0-45.6)1 | N/R | 73.8^a^ (49.8-109.2)2 | 67.5^a^ (40.7-112.0)3 | 38.2^a^ (9.5-152.7)1 |
|  | Stand Off | 52.4 (40.5-67.8)8 | 19.3^cde^ (10.4-35.8)1 | N/R | 76.7^ae^ (52.2-112.7)2 | 63.0^ae^ (37.3-106.4)4 | 305.4^acd^ (152.7-610.8)1 |
|  | Centre | 75.1 (64.5-87.4)7 | 28.9^cde^ (20.2-41.4)1 | N/R | 100.3^ae^ (79.1-127.3)2 | 128.3^a^ (99.0-166.3)3 | 210.0^ac^ (116.3-379.2)1 |
|  | Wing | 51.1 (42.5-61.4)8 | 13.5^cde^ (8.0-22.8)1 | N/R | 53.1^ade^ (38.3-73.6)2 | 121.6^ac^ (93.1-158.7)4 | 171.8^ac^ (89.4-330.2)1 |
|  | Fullback | 85.9 (70.3-105.0)8 | 44.3^cde^ (29.5-66.7)1 | N/R | 94.4^ad^ (66.8-133.5)2 | 157.6^ac^ (113.1-219.5)4 | 190.9^a^ (79.5-458.6)1 |
| **Player Group** | |  |  |  |  |  |  |
|  | Hit-Up-Forwards | 578.9 (531.5-630.5)9 | 33.3^cde^ (26.3-42.1)1 | N/R | 112.1^ade^ (95.7-131.5)2 | 236.0^ac^ (209.7-265.6)5 | 286.3^ac^ (200.2-409.5)1 |
|  | Outside-Backs | 530.5 (485.2-580.0)9 | 21.2^cde^ (15.8-28.5)1 | N/R | 76.7^ade^ (63.3-93.0)2 | 188.0^ac^ (164.6-214.6)5 | 190.9^ac^ (123.2-295.9)1 |
|  | Adjustables | 433.2 (396.5-473.2)9 | 30.1^cde^ (24.1-37.5)1 | N/R | 90.9^ade^ (77.6-106.4)2 | 169.6^ac^ (149.7-192.1)5 | 160.4^ac^ (104.6-245.9)1 |
| **Player Role** | |  |  |  |  |  |  |
|  | Forwards | 168.5g (162.4-174.9)18 | 77.4^bcdeg^ (71.6-83.8)5 | 91.9^acdeg^ (72.8-115.9)1 | 382.7^abdeg^ (361.1-405.6)3 | 198.2^abcg^ (186.1-211.2)8 | 235.4^abc^ (170.6-324.9)1 |
|  | Backs | 123.8f (119.0-128.8)18 | 53.1^cdef^ (48.6-57.9)5 | 42.1^cdef^ (30.7-57.9)1 | 276.9^abdef^ (259.9-294.9)3 | 158.3^abcf^ (148.3-169.1)8 | 185.4^abc^ (132.5-259.5)1 |

CI: Confidence Interval; n= number of studies; N/R = not reported; Significant difference (*p*<0.05) than (a) = Professional; (b) = Elite; (c) = Semi-Professional; (d) = Amateur; (e) = Junior; (f) = forwards; (g) = backs

**Supplementary Table 2:** Summary of pooled analysis of match injuries by total injuries recorded and participation level per 1,000 match-hrs. with 95% confidence intervals and number of studies by injury site for included published rugby league studies.

|  |  | **Total** | **Professional** | **Elite** | **Semi-Professional** | **Amateur** | **Junior** |
| --- | --- | --- | --- | --- | --- | --- | --- |
|  |  | **Rate (95% CI)n** | **Rate (95% CI)n** | **Rate (95% CI)n** | **Rate (95% CI)n** | **Rate (95% CI)n** | **Rate (95% CI)n** |
| **Head-Neck** | | **37.1 (35.7-38.6)32** | **29.1^bcde^ (27.4-30.8)9** | **7.4^acde^ (5.3-10.2)2** | **68.6^abde^ (63.0-74.7)5** | **53.3^abce^ (49.8-57.1)13** | **15.1^abcd^ (10.1-22.8)3** |
|  | Head | 28.3 (26.9-29.8)27 | 28.4^bde^ (26.5-30.5)8 | 6.6^acde^ (4.7-9.3)2 | 24.6^bde^ (20.8-29.0)4 | 49.1^abce^ (45.0-53.5)10 | 13.2^abcd^ (8.5-20.4)3 |
|  | Eye | 3.3 (1.7-6.4)2 | N/R | N/R | 3.0 (1.4-9.2)1 | N/R | 5.9 (1.5-23.5)1 |
|  | Nose | 2.6 (1.1-5.7)1 | N/R | N/R | 2.6 (1.1-5.7)1 | N/R | N/R |
|  | Face | 32.5 (29.8-35.5)8 | N/R | 1.2^cd^ (0.4-3.2)1 | 70.2^ade^ (63.5-77.6)4 | 21.8^ace^ (18.3-25.8)2 | 0.9^cd^ (0.1-6.5)1 |
|  | Mouth | 2.1 (0.9-5.1)1 | N/R | N/R | 2.1 (0.9-5.1)1 | N/R | N/R |
|  | Neck | 3.7 (2.0-6.9)2 | N/R | N/R | 2.6^e^ (1.1-5.7)1 | N/R | 11.7^c^ (4.4-31.3)1 |
| **Upper Limb** | | **32.1 (30.7-33.6)25** | **25.6^bcd^ (23.9-30.8)7** | **12.8^acde^ (10.0-16.3)2** | **72.8^abde^ (67.0-79.0)5** | **31.4^abc^ (28.5-34.7)9** | **23.7^bc^ (17.0-33.2)2** |
|  | Shoulder | 14.5 (13.4-15.8)20 | 10.9^cde^ (9.7-12.4)7 | 9.2^cde^ (6.9-12.2)2 | 31.9^abde^ (27.6-36.9)4 | 15.2^abc^ (12.2-19.0)6 | 19.5^abc^ (13.5-28.3)1 |
|  | Upper Arm | 4.3 (3.3-5.7)4 | 4.4 (3.4-5.7)3 | N/R | N/R | N/R | 2.9 (0.4-20.8)1 |
|  | Elbow | 2.4 (1.4-4.2)4 | N/R | 1.8 (0.6-5.6)1 | 3.0 (1.4-6.2)1 | 2.0 (0.5-7.8)1 | 2.9 (0.4-20.8)1 |
|  | Lower Arm | 23.7 (21.5-26.0)10 | 3.0^bc^ (1.8-5.3)2 | 4.5^ac^ (2.7-7.4)1 | 65.8^abde^ (59.5-72.8)4 | 4.1^c^ (2.4-7.0)2 | 3.7^c^ (1.4-9.8)1 |
|  | Wrist | 1.6 (0.9-2.9)4 | 1.0^e^ (0.3-3.0)1 | N/R | 1.7 (0.6-4.5)1 | 2.9 (0.9-9.1)1 | 2.9^a^ (0.4-20.8)1 |
|  | Hand | 4.6 (3.4-6.2)5 | 3.2 (1.7-5.9)1 | 6.0 (3.2-11.1)1 | 5.0 (3.3-7.6)2 | N/R | 5.9 (1.5-23.5)1 |
|  | Finger | 1.5 (0.6-4.0)2 | N/R | N/R | 0.9^e^ (0.2-3.4)1 | N/R | 5.9^c^ (1.5-23.5)1 |
| **Lower Limb** | | **64.5 (62.5-66.7)25** | **54.3^bcde^ (51.7-56.9)7** | **27.3^acde^ (23.1-32.3)2** | **157.2^abde^ (148.7-166.3)5** | **50.0^abc^ (46.2-54.1)9** | **38.4^abc^ (29.5-50.0)2** |
|  | Quadriceps | 28.5 (26.4-30.8)12 | 15.5^c^ (13.1-18.4)3 | 11.7^ce^ (8.5-16.0)1 | 85.5^abde^ (77.5-94.4)3 | 14.1^c^ (11.0-18.0)3 | 4.9^bc^ (2.3-10.3)2 |
|  | Hamstrings | 7.0 (5.4-9.1)4 | 1.6^bc^ (0.7-3.8)1 | 7.2^ac^ (4.1-12.6)1 | 17.5^abd^ (12.6-24.3)1 | 2.0^c^ (0.5-7.8)1 | N/R |
|  | Knee | 22.3 (21.0-23.7)23 | 16.8^bc^ (15.2-18.5)7 | 7.6^acde^ (5.5-10.4)2 | 54.7^abde^ (49.7-60.2)5 | 18.1^bc^ (15.8-20.8)7 | 19.5^bc^ (13.5-28.3)2 |
|  | Lower Leg | 24.2 (21.5-27.2)6 | 10.5^c^ (7.9-13.8)1 | 7.2^c^ (4.1-12.6)1 | 59.1^abde^ (51.4-67.9)2 | 5.9^c^ (2.6-13.1)1 | 11.7^c^ (4.4-31.3)1 |
|  | Ankle | 12.1 (11.0-13.2)21 | 9.7^c^ (8.5-11.0)7 | 7.2^c^ (5.2-10.0)2 | 28.1^abde^ (24.0-32.8)4 | 10.0^c^ (7.6-13.2)6 | 11.2^c^ (6.8-18.2)2 |
|  | Foot | 1.5 (0.8-2.9)3 | 1.3 (0.5-3.4)1 | N/R | 2.1 (0.9-5.1)1 | 1.0 (0.1-6.9)1 | N/R |
| **Chest-Back-Other** | | **15.9 (14.9-17.0)26** | **15.4^bde^ (26.5-30.5)8** | **5.2^ace^ (3.5-7.6)2** | **17.8^be^ (15.1-21.0)5** | **20.8^ae^ (18.4-23.5)9** | **11.2^abcd^ (6.8-18.2)2** |
|  | Spine | 4.4 (2.9-6.5)2 | 4.1 (2.4-7.1)1 | N/R | 4.7 (2.6-8.4)1 | N/R | N/R |
|  | Lower Back | 4.5 (2.5-7.8)2 | N/R | 4.8 (2.4-9.6)1 | N/R | 3.9 (1.5-10.4)1 | N/R |
|  | Chest | 7.7 (6.1-9.8)4 | 7.9^e^ (6.1-10.2)2 | N/R | N/R | 2.9^e^ (0.9-9.1)1 | 17.6^ad^ (7.9-39.2)1 |
|  | Sternum | 5.4 (3.9-7.6)3 | N/R | 7.2 (4.1-12.6)1 | 4.8 (3.1-7.3)2 | N/R | N/R |
|  | Abdomen | 16.0 (14.3-17.9)9 | 9.3^bcde^ (7.2-12.1)2 | 3.2^acde^ (2.0-5.2)2 | 96.8^abde^ (79.5-117.7)1 | 21.2^abce^ (17.8-25.3)2 | 7.3^abcd^ (3.7-14.6)1 |
|  | Pelvis | 5.1 (3.1-8.3)1 | 5.1 (3.1-8.3)1 | N/R | N/R | N/R | N/R |
|  | Genitals | 2.6 (1.2-5.5)2 | N/R | N/R | 2.6 (1.1-5.7)1 | N/R | 2.9 (0.4-20.8)1 |
|  | Other | 5.3 (4.3-6.6)6 | 9.2^bc^ (7.0-12.2)2 | 2.0^acde^ (1.1-3.7)1 | 4.9^ab^ (2.6-9.0)1 | 14.2^b^ (2.0-100.6)1 | 6.4^b^ (3.1-13.4)1 |

CI: Confidence Interval; n= number of studies; N/R = not reported; Significant difference (*p*<0.05) than (a) = Professional; (b) = Elite; (c) = Semi-Professional; (d) = Amateur; (e) = Junior

**Supplementary Table 3:** Summary of pooled analysis of match injuries by total injuries recorded and participation level per 1,000 match-hrs. with 95% confidence intervals and number of studies by injury type and match period for included published rugby league studies.

|  |  | **Total** | **Professional** | **Elite** | **Semi-Professional** | **Amateur** | **Junior** |
| --- | --- | --- | --- | --- | --- | --- | --- |
|  |  | **Rate (95% CI)n** | **Rate (95% CI)n** | **Rate (95% CI)n** | **Rate (95% CI)n** | **Rate (95% CI)n** | **Rate (95% CI)n** |
| **Injury type** | |  |  |  |  |  |  |
|  | Haematomas | 42.9 (40.5-45.4)12 | 9.8^bcde^ (8.0-12.1)2 | 12.2^acd^ (9.5-15.6)2 | 137.5^abde^ (127.7-148.0)3 | 44.7^abce^ (11.5-26.5)3 | 14.0^acd^ (9.0-21.6)2 |
|  | Contusion | 38.7 (37.0-40.6)20 | 21.5^bcde^ (19.9-23.3)7 | 8.4^acd^ (5.8-12.1)1 | 80.5^abde^ (74.3-87.2)3 | 100.0^abce^ (91.9-108.9)7 | 5.6^acd^ (2.8-11.2)2 |
|  | Strain | 31.7 (30.5-33.0)26 | 20.5^bcde^ (19.3-21.7)9 | 9.2^acd^ (6.9-12.2)2 | 110.0^abde^ (102.7-117.7)4 | 44.3^abce^ (40.7-48.1)9 | 11.2^acd^ (6.8-18.2)2 |
|  | Sprain | 28.4 (27.2-29.6)27 | 22.2^bcde^ (21.0-23.5)9 | 18.5^acde^ (15.1-22.7)2 | 69.7^abde^ (63.9-75.9)4 | 33.5^abc^ (30.4-36.8)10 | 32.1^abc^ (24.1-42.9)2 |
|  | Fracture-Dislocations | 21.9 (20.5-23.4)24 | 11.6^bd^ (10.7-12.6)9 | 6.2^acde^ (4.3-8.8)2 | 13.4^bd^ (10.7-16.9)3 | 18.5^abc^ (15.3-22.3)8 | 12.6^b^ (12.67.9-19.9)2 |
|  | Abrasion | 11.4 (9.9-13.1)9 | 2.6^c^ (1.5-4.4)2 | 0.4^cd^ (0.1-1.6)2 | 55.4^abde^ (47.7-64.3)2 | 2.4^bc^ (1.2-4.7)2 | 2.9^c^ (0.4-20.8)1 |
|  | Lacerations | 12.8 (11.9-13.9)21 | 12.8^bde^ (11.6-14.2)6 | 3.0^acd^ (1.8-5.0)2 | 11.8^bde^ (9.2-15.0)3 | 19.0^abce^ (16.6-21.7)8 | 3.5^acd^ (1.5-8.4)2 |
|  | Concussion | 8.9 (8.2-9.5)28 | 8.8^b^ (8.1-9.6)10 | 3.6^acde^ (2.3-5.7)2 | 10.7^a^ (8.2-13.8)3 | 11.1^a^ (8.9-13.7)10 | 11.2^a^ (7.0-18.0)3 |
|  | Other | 6.4 (5.4-7.6)12 | 1.7^bcde^ (0.9-3.2)2 | 5.0^ac^ (3.4-7.4)2 | 15.5^abde^ (11.7-20.6)2 | 7.0^ac^ (5.2-9.6)5 | 7.3^ac^ (3.7-14.6)1 |
| **Injury Cause** | |  |  |  |  |  |  |
|  | Tackler | 36.3 (34.5-38.3)25 | 14.2^cd^ (12.6-15.9)7 | 13.2^cd^ (9.8-17.7)1 | 78.2^abe^ (72.2-84.6)5 | 82.7^abe^ (75.1-91.0)10 | 13.6^cd^ (8.3-22.1)2 |
|  | Ball Carrier | 58.2 (55.8-60.7)25 | 24.6^cd^ (22.6-26.9)7 | 16.5^cd^ (12.6-21.4)1 | 117.4^ade^ b(110.0-125.3)5 | 140.3^abce^ (130.3-151.1)10 | 19.5^cd^ (13.0-29.3)2 |
|  | Contact | 17.2 (13.7-21.7)3 | N/R | N/R | 20.7 (16.2-26.5)2 | N/R | 7.3 (3.7-14.6)1 |
|  | Collision Player | 35.8 (32.7-39.2)10 | N/R | 3.6^cde^ (2.0-3.3)1 | 55.1^bde^ (50.1-60.6)5 | 16.5^bc^ (9.4-29.0)3 | 10.1^bc^ (5.6-1.8)1 |
|  | Collision Other | 25.8 (23.1-28.7)8 | N/R | 3.3^c^ (1.8-5.9)1 | 41.5^bde^ (37.2-46.3)5 | 2.2^c^ (0.6-8.9)1 | 1.8^c^ (0.5-7.3)1 |
|  | Fall | 12.9 (10.6-15.6)6 | N/R | 5.4^cd^ (3.4-8.6)1 | 22.0^be^ (17.6-27.3)3 | 28.3^be^ (7.1-113.3)1 | 4.6^cd^ (1.9-11.0)1 |
|  | Slip | 0.4 (0.1-3.0)1 | N/R | N/R | 0.4 (0.1-3.0)1 | N/R | N/R |
|  | Twist | 1.9 (1.1-3.3)4 | N/R | 0.9^d^ (0.3-2.8)1 | 3.0^d^ (1.4-6.2)1 | 28.3^bce^ (7.1-113.3)1 | 0.9^d^ (0.1-6.5)1 |
|  | Overexertion | 14.3 (12.0-16.9)6 | N/R | 3.9^cd^ (2.3-6.7)1 | 20.2^b^ (16.8-24.2)4 | 28.3^b^ (7.1-113.3)1 | N/R |
|  | Overuse | 3.2 (2.2-4.6)6 | N/R | 1.2^cd^ (0.4-3.2)1 | 4.0^bd^ (2.7-6.1)4 | 28.3^bc^ (7.1-113.3)1 | N/R |
|  | Other | 18.7 (17.1-20.6)15 | 23.6^bce^ (20.8-26.7)5 | 7.5^ad^ (5.1-11.1)1 | 7.0^ad^ (5.1-9.6)4 | 31.7^bce^ (26.3-38.3)4 | 8.2^ad^ (4.3-15.8)1 |
| **Injury Severity** | |  |  |  |  |  |  |
|  | Transient | 114.7 (112.2-117.2)21 | 70.3^bcd^ (68.2-72.5)9 | 32.5^acde^ (24.6-42.8)1 | 485.1^abde^ (467.3-503.5)4 | 382.5^abce^ (358.2-408.5)6 | 88.1^bcd^ (61.6-126.0)1 |
|  | Mild | 34.9 (32.8-37.2)16 | 17.0^bcde^ (15.2-19.0)5 | 176.0^acde^ (156.2-198.2)1 | 30.7^abde^ (26.4-35.7)3 | 87.5^abc^ (76.3-100.4)6 | 85.2^abc^ (59.2-122.6)1 |
|  | Moderate | 8.6 (7.9-9.3)19 | 4.6^bcde^ (4.0-5.2)8 | 31.2^a^ (23.5-41.4)1 | 31.4^a^ (27.1-36.5)3 | 30.4^a^ (24.1-38.4)6 | 32.3^a^ (17.9-58.3)1 |
|  | Major | 13.4 (12.2-14.6)23 | 8.6^bcde^ (7.5-9.9)7 | 3.2^acde^ (1.4-7.8)1 | 19.1^abde^ (15.9-23.1)4 | 32.7^abc^ (27.8-38.3)9 | 25.7^abc^ (14.2-46.4)2 |
| **Match Period** | |  |  |  |  |  |  |
|  | 1st quarter | 95.6 (79.6-114.9)7 | N/R | N/R | 57.8^de^ (41.3-80.9)1 | 93.9^c^ (77.5-113.9)5 | 117.5^c^ (63.2-218.3)1 |
|  | 2nd quarter | 260.0 (232.7-290.7)7 | N/R | N/R | 120.8^de^ (95.7-152.4)1 | 256.6^c^ (228.4-288.2)5 | 305.4^c^ (208.0-448.6)1 |
|  | 3rd quarter | 261.7 (234.2-292.4)7 | N/R | N/R | 161.6^de^ (132.2-197.6)1 | 260.2^c^ (231.8-292.0)5 | 281.9^c^ (189.0-420.6)1 |
|  | 4th quarter | 287.7 (258.8-319.8)7 | N/R | N/R | 107.2^d^ (83.7-137.2)1 | 297.2^cd^ (266.8-186.7)5 | 164.5^d^ (97.4-277.7)1 |
|  | 1st half | 161.7^i^ (152.4-171.5)11 | N/R | N/R | 90.2^dei^ (74.5-109.1)1 | 175.3^ci^ (164.6-186.7)9 | 211.5^c^ (152.5-293.1)1 |
|  | 2nd half | 183.3^h^ (173.5-193.7)11 | N/R | N/R | 139.5^deh^ (119.7-162.6)1 | 191.4^ch^ (180.2-203.3)9 | 223.2^c^ (162.4-306.7)1 |

CI: Confidence Interval; n= number of studies; N/R = not reported; Significant difference (*p*<0.05) than (a) = Professional; (b) = Elite; (c) = Semi-Professional; (d) = Amateur; (e) = Junior; (f) = forwards; (g) = backs; (h) = 1^st^ half; (i) = 2^nd^ half

**Supplementary Table 4:** Summary of pooled analysis of training injuries by total and participation level per 1,000 training-hrs. with 95% confidence intervals and number of studies by player role, injury site and injury type for included published rugby league studies.

|  |  | **Total** | **Professional** | **Elite** | **Semi-Professional** | **Amateur** |
| --- | --- | --- | --- | --- | --- | --- |
|  |  | **Rate (95% CI)n** | **Rate (95% CI)n** | **Rate (95% CI)n** | **Rate (95% CI)n** | **Rate (95% CI)n** |
| **Player Role** | |  |  |  |  |  |
|  | Forwards | 13.9^f^ (12.9-15.1)4 | N/R | N/R | 25.9^df^ (23.2-28.9)1 | 3.2^c^ (2.6-3.9)3 |
|  | Backs | 10.6^e^ (9.7-11.7)4 | N/R | N/R | 19.3^de^ (17.0-22.0)1 | 2.6^c^ (2.1-3.2)3 |
| **Head-Neck** | | **1.2 (1.1-1.4)10** | **0.3^bcd^ (0.2-0.4)2** | **8.8^acd^ (7.2-10.8)2** | **1.8^abd^ (1.3-2.4)3** | **0.7^abc^ (0.5-1.1)3** |
|  | Head | 1.1 (0.9-1.2)8 | 0.3^bcd^ (0.2-0.4)2 | 3.0^ad^ (2.1-4.3)2 | 1.7^ad^ (1.1-2.8)2 | 0.7^abc^ (0.4-1.1)2 |
|  | Face | 4.1 (3.3-5.0)4 | N/R | 5.8^c^ (4.5-7.4)2 | 2.2^b^ (1.5-3.4)2 | N/R |
| **Upper Limb** | | **1.2 (1.1-1.3)9** | **0.2^bcd^ (0.2-0.3)2** | **9.6^acd^ (7.9-11.7)2** | **2.4^ab^ (1.9-3.2)3** | **2.8^ab^ (2.0-3.9)2** |
|  | Shoulder | 0.5 (0.4-0.6)8 | 0.1^bcd^ (0.1-0.2)3 | 2.6^a^ (1.8-3.7)2 | 1.7^a^ (1.1-2.8)2 | 1.3^a^ (0.3-5.3)1 |
|  | Upper Arm | 8.6 (6.1-11.9)1 | N/R | 8.6 (6.1-11.9)1 | N/R | N/R |
|  | Elbow | 0.3 (0.0-2.5)1 | 0.3 (0.0-2.5)1 | N/R | N/R | N/R |
|  | Lower Arm | 4.6 (3.7-5.8)3 | N/R | 6.1^c^ (4.4-8.3)1 | 3.7^b^ (2.7-5.2)2 | N/R |
|  | Hand | 0.3 (0.1-1.3)1 | 0.3 (0.1-1.3)1 | N/R | N/R | N/R |
| **Lower Limb** | | **8.4 (8.1-8.7)9** | **10.6^bc^ (9.4-11.9)2** | **65.8^acd^ (61.1-70.9)2** | **36.2^abd^ (33.7-38.7)3** | **8.6^bc^ (7.2-10.4)2** |
|  | Quadriceps | 11.7 (10.9-12.6)6 | 1.5^bc^ (1.2-2.0)2 | 31.5^ac^ (28.3-35.1)2 | 15.6^ab^ (13.9-17.5)2 | N/R |
|  | Hamstrings | 0.8 (0.6-1.3)1 | 0.8 (0.6-1.3)1 | N/R | N/R | N/R |
|  | Knee | 1.5 (1.4-1.7)10 | 0.4^bcd^ (0.3-0.5)4 | 12.0^ac^ (10.1-14.3)2 | 6.0^ab^ (5.1-7.1)3 | 7.3^a^ (4.1-13.2)1 |
|  | Lower Leg | 4.4 (3.8-5.1)4 | 0.8^c^ (0.5-1.2)3 | N/R | 38.6^a^ (32.8-45.6)1 | N/R |
|  | Ankle | 2.8 (2.6-3.0)9 | 0.4^bcd^ (0.3-0.5)3 | 22.3^acd^ (19.6-25.4)2 | 10.7^abd^ (9.5-12.2)3 | 3.3^abc^ (1.4-8.0)1 |
| **Chest-Back-Other** | | **1.8 (1.6-2.0)9** | **0.7^bc^ (0.6-0.8)2** | **12.8^acd^ (10.8-15.2)2** | **6.0^abd^ (5.0-7.1)3** | **0.8^bc^ (0.4-1.4)2** |
|  | Abdomen | 4.2 (3.7-4.7)8 | 0.7^bc^ (0.5-1.1)3 | 12.0^ac^ (10.1-14.3)2 | 5.9^ab^ (5.0-7.0)3 | N/R |
|  | Other | 1.6 (1.2-2.3)6 | 1.1 (0.5-2.4)1 | 0.9 (0.4-1.6)2 | 0.3 (0.0-1.9)1 | 0.8 (0.4-1.4)2 |
| **Injury type** | |  |  |  |  |  |
|  | Abrasion | 8.4 (7.2-9.7)4 | N/R | 11.5^c^ (9.6-13.7)2 | 4.3^b^ (3.2-5.8)2 | N/R |
|  | Blister | 6.6 (5.3-8.4)2 | N/R | 6.6 (5.3-8.4)2 | N/R | N/R |
|  | Strain | 4.4 (4.2-4.7)10 | 0.8^bcd^ (0.7-0.9)3 | 25.9^ab^ (23.0-29.2)2 | 23.2^ad^ (21.3-25.3)3 | 5.0^abc^ (3.9-6.3)2 |
|  | Haematomas | 4.3 (3.5-5.2)5 | 0.3^bc^ (0.0-2.5)1 | 3.4^a^ (2.5-4.7)2 | 2.6^a^ (1.8-3.9)2 | N/R |
|  | Other | 4.2 (3.7-4.9)7 | 3.6^bcd^ (2.5-5.0)2 | 7.3^ad^ (5.8-9.1)2 | 6.1^ad^ (4.7-7.8)2 | 0.1^abc^ (0.0-0.6)1 |
|  | Sprain | 3.4 (3.2-3.6)11 | 1.1^bcd^ (1.0-1.2)4 | 29.0^acd^ (25.9-32.4)2 | 9.7^abd^ (8.4-11.0)3 | 2.3^abc^ (1.6-3.3)2 |
|  | Contusion | 1.4 (1.3-1.6)9 | 0.2^bcd^ (0.1-0.3)2 | 10.5^acd^ (8.8-12.7)2 | 5.0^abd^ (4.2-6.1)3 | 1.5^abc^ (1.0-2.4)2 |
|  | Lacerations | 0.6 (0.5-0.7)4 | 0.1^b^ (0.1-0.2)2 | 1.2^a^ (0.7-2.1)2 | N/R | N/R |
|  | Fracture-Dislocations | 0.3 (0.2-0.4)7 | 0.1^bcd^ (0.0-0.1)2 | 1.1^ac^ (0.6-2.0)2 | 0.4^ab^ (0.2-1.1)2 | 0.7^a^ (0.3-1.4)1 |
|  | Concussion | 0.1 (0.0-0.1)6 | 0.01^bcd^ (0.00-0.04)1 | 0.5^a^ (0.2-1.1)2 | 0.3^a^ (0.0-1.9)1 | 0.2^a^ (0.1-0.7)2 |

CI: Confidence Interval; n= number of studies; N/R = not reported; Significant difference (*p*<0.05) than (a) = Professional; (b) = Elite; (c) = Semi-Professional; (d) = Amateur; (e) = Forwards; (f) = Backs

**Supplementary Table 5:** Summary of pooled analysis of training injuries by total and participation level per 1,000 training-hrs. with 95% confidence intervals and number of studies by injury cause, injury severity and training period for included published rugby league studies.

|  |  | **Total** | **Professional** | **Elite** | **Semi-Professional** | **Amateur** |
| --- | --- | --- | --- | --- | --- | --- |
|  |  | **Rate (95% CI)n** | **Rate (95% CI)n** | **Rate (95% CI)n** | **Rate (95% CI)n** | **Rate (95% CI)n** |
| **Injury Cause** | |  |  |  |  |  |
|  | Overexertion | 17.9 (16.6-19.3)5 | N/R | 33.4^cd^ (30.1-37.1)2 | 20.5^bd^ (18.4-22.8)2 | 0.1^bc^ (0.0-0.6)1 |
|  | Contact | 13.9 (10.5-18.3)1 | N/R | N/R | 13.9 (10.5-18.3)1 | N/R |
|  | Collision Other | 8.6 (7.2-10.3)3 | N/R | 8.7 (7.1-10.7)2 | 8.2 (5.7-11.7)1 | N/R |
|  | Fall | 8.3 (7.3-9.5)4 | N/R | 10.6^c^ (8.8-12.8)2 | 6.8^d^ (5.6-8.2)2 | N/R |
|  | Collision Player | 8.2 (7.2-9.4)4 | N/R | 17.3^cd^ (14.9-20.0)2 | 3.3^bd^ (1.9-5.8)1 | 1.5^bc^ (0.9-2.4)1 |
|  | Overuse | 6.9 (6.2-7.8)4 | 0.4^bc^ (0.2-0.7)1 | 18.8^a^ (16.4-21.6)2 | 19.6^a^ (15.6-24.7)1 | N/R |
|  | Other | 6.3 (5.5-7.3)5 | N/R | 4.3^cd^ (3.2-5.7)2 | 1.6^b^ (0.7-3.6)1 | 1.9^b^ (1.4-2.4)2 |
|  | Ball Carrier | 1.2 (1.0-1.3)7 | 0.3^bcd^ (0.2-0.4)1 | 2.4^ad^ (1.6-3.5)2 | 0.8^a^ (0.3-2.5)1 | 0.9^ac^ (0.6-1.3)3 |
|  | Tackler | 0.8 (0.7-0.9)6 | 0.2^bcd^ (0.2-0.3)1 | 1.5^acd^ (0.9-2.5)2 | 0.5^abd^ (0.1-2.2)1 | 2.9^abc^ (2.1-4.0)2 |
|  | Slip | 0.3 (0.0-1.9)1 | N/R | N/R | 0.3 (0.0-1.9)1 | N/R |
| **Injury Severity** | |  |  |  |  |  |
|  | Transient | 9.1 (8.8-9.5)9 | 2.4^bcd^ (2.2-2.6)3 | 83.4^acd^ (75.0-92.8)1 | 46.7^abd^ (43.9-49.6)3 | 4.6^abc^ (3.6-5.9)2 |
|  | Mild | 1.7 (1.5-1.9)7 | 0.5^bcd^ (0.4-0.6)2 | 24.2^acd^ (19.9-29.5)1 | 3.1^abd^ (2.7-4.0)2 | 6.1^abc^ (4.9-7.6)2 |
|  | Moderate | 0.4 (0.3-0.5)8 | 0.2^bcd^ (0.2-0.3)2 | 2.4^acd^ (1.3-4.5)1 | 0.5^ab^ (0.3-0.9)3 | 1.0^ab^ (0.6-1.7)2 |
|  | Major | 0.3 (0.3-0.4)8 | 0.1^d^ (0.1-0.2)2 | 0.5 (0.1-2.0)1 | 0.3^d^ (0.1-0.6)3 | 0.6^ac^ (0.4-0.9)2 |
| **Training Period** | |  |  |  |  |  |
|  | 1st quarter | 1.7 (1.0-3.0)1 | N/R | N/R | N/R | 1.7 (1.0-3.0)1 |
|  | 2nd quarter | 4.0 (2.8-5.7)2 | N/R | N/R | N/R | 4.0 (2.8-5.7)2 |
|  | 3rd quarter | 5.6 (4.2-7.6)2 | N/R | N/R | N/R | 5.6 (4.2-7.6)2 |
|  | 4th quarter | 4.2 (2.9-5.9)2 | N/R | N/R | N/R | 4.2 (2.9-5.9)2 |
|  | 1st half | 4.9f (3.9-6.1)3 | N/R | N/R | N/R | 4.9^f^ (3.9-6.1)3 |
|  | 2nd half | 6.7e (5.5-8.1)3 | N/R | N/R | N/R | 6.7^e^ (5.5-8.1)3 |

CI: Confidence Interval; n= number of studies; N/R = not reported; Significant difference (*p*<0.05) than (a) = Professional; (b) = Elite; (c) = Semi-Professional; (d) = Amateur; (e) = 1^st^ half; (f) = 2^nd^ half
